# *Lgr5*^+^ cell fate regulation by coordination of metabolic nuclear receptors during liver repair

**DOI:** 10.7150/thno.74194

**Published:** 2022-08-15

**Authors:** Dan Qin, Shenghui Liu, Yuanyuan Lu, Yi Yan, Jing Zhang, Shiyao Cao, Mi Chen, Ning Chen, Wendong Huang, Liqiang Wang, Xiangmei Chen, Lisheng Zhang

**Affiliations:** 1 College of Veterinary Medicine/College of Biomedicine and Health, Huazhong Agricultural University, Wuhan, Hu Bei, 430070, China.; 2 Department of Diabetes Complications and Metabolism, Diabetes and Metabolism Research Institute, Beckman Research Institute, City of Hope National Medical Center, Duarte, CA 91010, USA.; 3 Department of Nephrology, First Medical Center of Chinese PLA General Hospital, Nephrology Institute of the Chinese People's Liberation Army, State Key Laboratory of Kidney Diseases, National Clinical Research Center for Kidney Diseases, Beijing Key Laboratory of Kidney Disease Research, Beijing 100853, China.

**Keywords:** FXR, PPARα, *Lgr5*, Proliferation, Symmetric or asymmetric cell division

## Abstract

**Background:** Leucine-rich repeat-containing G protein-coupled receptor 5 (*Lgr5*) is a target gene of Wnt/β-Catenin which plays a vital role in hepatic development and regeneration. However, the regulation of *Lgr5* gene and the fate of *Lgr5*^+^ cells in hepatic physiology and pathology are little known. This study aims to clarify the effect of metabolic nuclear receptors on *Lgr5*^+^ cell fate in liver.

**Methods:** We performed cell experiments with primary hepatocytes, Hep 1-6, Hep G2, and Huh 7 cells, and animal studies with wild-type (WT), farnesoid X receptor (FXR) knockout mice, peroxisome proliferator-activated receptor α (PPARα) knockout mice and *Lgr5*-Cre^ERT2^; Rosa26-mTmG mice. GW4064 and CDCA were used to activate FXR. And GW7647 or Wy14643 was used for PPARα activation. Regulation of *Lgr5* by FXR and PPARα was determined by QRT-PCR, western blot (WB) and RNAscope^®^ *in situ* hybridization (ISH) and immunofluorescence (IF), luciferase reporter assay, electrophoretic mobility shift assay (EMSA) and chromatin immunoprecipitation (ChIP). Diethyl 1,4-dihydro-2,4,6-trimethyl-3,5-pyridinedicarboxylate (DDC) diet was used to induce liver injury.

**Results:** Pharmacologic activation of FXR induced *Lgr5* expression, whereas activation of PPARα suppressed *Lgr5* expression. Furthermore, FXR and PPARα competed for binding to shared site on *Lgr5* promoter with opposite transcriptional outputs. DDC diet triggered the transition of *Lgr5*^+^ cells from resting state to proliferation. FXR activation enhanced *Lgr5*^+^ cell expansion mainly by symmetric cell division, but PPARα activation prevented *Lgr5*^+^ cell proliferation along with asymmetric cell division.

**Conclusion:** Our findings unravel the opposite regulatory effects of FXR and PPARα on *Lgr5*^+^ cell fate in liver under physiological and pathological conditions, which will greatly assist novel therapeutic development targeting nuclear receptors.

## Introduction

The liver possesses excellent regenerative ability and exerts multiple physiological functions. Insufficient liver regeneration leads to liver failure 1. In addition, chronic liver disease may evoke over-proliferation of cells to induce liver neoplasia 2, 3. Therefore, exploring the cell source for newly generated hepatocytes after injury and the regulatory mechanism underlying the initiation and termination of hepatocyte proliferation should be the research focus.

*Lgr5* is a target gene of Wnt/β-catenin and is widely known as an important tissue stem cell marker 4. Studies showed that small *Lgr5*-LacZ cells appear around near the bile ducts when the liver is injured 5. And *Lgr5*^+^ cells isolated from injured liver could be clonally expanded into organoids *in vitro* and differentiated to functional hepatocytes *in vivo* 5. Likewise, another study showed that carbon tetrachloride treatment promotes *Lgr5*^+^ liver stem cell proliferation and improves liver fibrosis, whereas *Lgr5* knockdown worsens fibrosis 6. Besides, human liver bile duct-derived *Lgr5*^+^ bi-potent progenitor cells after prolonged culture are highly stable at the chromosome structural level 7. These results suggested that *Lgr5*^+^ cells are beneficial to liver regeneration.

However, other studies suggest that *Lgr5*^+^ cells induce the occurrence and development of hepatocellular carcinoma (HCC) 8, 9. *Lgr5*^+^ cells are functionally similar to the cancer initiating cells, and they are capable of forming tumors and resisting chemotherapeutic drugs 9. A clinical study has reported that 77 out of 188 HCC specimens showed positive staining for *Lgr5*, and the occurrence frequency of *Lgr5* in tumor area was remarkably higher than that in the non-tumor area. Furthermore, *Lgr5* expression and HCC progression showed a positive correlation 10. Importantly, *Lgr5* deletion notably impedes initiation of organoid and tumor 10. Since *Lgr5^+^* cells may be a double-edged sword for liver injury and repair, it is particularly important to reveal the spatial and temporal regulatory mechanism of *Lgr5*.

FXR regulates *Lgr5*^+^ intestinal stem cell proliferation 11. FXR, as a transcription factor, is activated by a specific ligand such as bile acids (BA) 12. Both hepatic FXR and intestinal FXR promote liver regeneration/repair, alleviate liver regeneration defect, and prevent cell death 13. Recently, glutamine metabolism has been found to promote activation of Wnt signaling, increase expression of *Lgr5*, and enhance functions of stem cell 14. Furthermore, FXR controls amino acid catabolism and ammonium detoxification through ureagenesis and glutamine synthesis in liver 15. It has been reported that PPAR activation in intestinal crypts suppresses Wnt signal and promotes the loss of stemness of the *Lgr5*^+^ stem cells 16. Besides, PPARα, belonging to the PPARs, regulates multiple biological processes 17. PPARα is a major inducer of hepatic oxidative phosphorylation 18 which is important for stem cell fate in various tissues 19. And both of FXR and PPARα can bind to the same binding sites using RXR as cofactor. However, whether FXR or PPARα regulates *Lgr5* in the liver has not been reported yet. Understanding the regulatory mechanisms by which liver stem cells utilize metabolic nuclear receptors will disclose how liver maintain homeostasis and injury repair.

In this study, we further explored the response of *Lgr5 cells* to liver damage, and the regulation of *Lgr5* cell fate by metabolic nuclear receptors.

## Methods

All animal studies and procedures followed the guidelines for the care and use of laboratory animals of Huazhong Agricultural University. FXR knockout (KO) mice (# 007214) and PPARα knockout mice (# 008154) were brought from Jackson Laboratory (JAX, USA). *Lgr5*-Cre^ERT2^; Rosa26-mTmG mice (EGE-WXH-042) were bought from Biocytogen (Beijing, China). Mice were administrated with either vehicle (80% PEG-400 and 20% Tween 80), or GW4064 (MCE, HY50108, 50 mg/kg body weight), or GW7647 (Cayman, 265129-71-3, 5 mg/kg body weight) or Wy14643 (Cayman, 50892-23-4,100 mg/kg body weight) twice a day for 2 consecutive days, if no otherwise specified 20. Mice were administrated with either vehicle or CDCA (Sigma, 700198P, 20 mg/kg body weight) once per day for 7 consecutive days 21. For DDC injury experiments, 6-8 week (wk) old mice were fed with 0.1% DDC diet for 7 days 22. For lineage tracing, heterozygous *Lgr5*-Cre^ERT2^; Rosa26-mTmG mice were intraperitoneally injected with a single dose of tamoxifen (Sigma, T5648, 100 mg/kg body weight/day) once per day for 3 days and stop injection for 7 days before treatment. BrdU (Sigma, B5002, 50 mg/kg) was injected twice per day for 2 days before sacrifice. Mice were anesthetized to obtain blood and livers. For EdU (Servicebio, G5059) labeling, mice were injected with EdU (5 mg/kg) for 4 h or 24 h and then sacrificed to collect liver samples and processed for EdU detection according to the protocol of the manufacturer (Servicebio, G1601).

### Cell culture

Primary hepatocytes were isolated by collagenase perfusion of the liver according to Taniai with minor modification 23. Primary hepatocytes, Hep G2, Hep 1-6, and Huh 7 cells were incubated in H-DMEM (Thermo Fisher, Waltham, USA) with 10% fetal bovine serum (FBS, Thermo Fisher, Waltham, USA) and 100 IU/mL penicillin-streptomycin (Hyclone, sv30010). All the cells were cultured in a humidified incubator containing 5% CO_2_ at 37 °C. Cells were treated with GW4064 (10 μM), GW7647 (10 μM), Wy14643 (100 μM) or MK886 (TOPSCIENCE, T6893, 5 μM) for 24 h prior to RNA isolation 20, 22, 24, 25. In order to detect the specificity of agonists, primary hepatocytes were isolated from WT, FXR KO or PPARα KO mice and then co-treated with different dose of GW4064 (1.0 μM or 10 μM) and GW7647 (0.1 μM or 1.0 μM) for 24 h 20, 26-28.

### QRT-PCR

By using RNAiso plus (Takara, Japan), total RNA was isolated and purified, and the first strand cDNA synthesis kit (TOYOBO, Japan) was used to transcribe the obtained RNA into cDNA. The cDNA was diluted and used for the QRT-PCR. In a 10 μL reaction system, the samples were run with the SYBR Green QRT-PCR mix (TOYOBO, Japan). Data were analyzed in the ABI CFX Connect TM Real-Time PCR Detection System (ABI, USA). The mRNA level of the interest gene was normalized to housekeeping gene *36B4* or *Gapdh*. The primers were listed in Table S1.

### Western blot

Mouse livers and primary hepatocytes were extracted with protein lysis buffer (Beyotime, China, P0013) supplemented with protease inhibitor cocktail. BCA Kit (Beyotime, China, P0009) was used to assess the protein concentration. Proteins (40 µg) were separated on a 10% or 4-20% polyacrylamide precast SDS gel followed by blotting on PVDF membranes (Millipore Billerica, MA, USA). The membranes were probed with the following antibodies against: anti-LGR5 (Thermofisher, MA5-25644), anti-SHP (Abclone, A16454), anti-CPT1a (Santa Cruz, sc-393070) and anti-GAPDH (Santa Cruz, sc-293335) at 4 °C overnight. The secondary antibody was incubated at the dilution concentration of 1:5,000 for 1.5 h at room temperature. Then the membrane was exposed with ECL reagent (Juneng, K-12045-D10) in the imaging system (SYNGENE, G: Box). The density of the bands was analyzed by using Image-J software.

### Molecular cloning and cell-based luciferase reporter assay

Putative FXREs and PPREs in *Lgr5* promoter sequence were analyzed using an online algorithm (NUBIScan). According to this prediction, the gene promoter fragments (position -3332 to -1824, -1994 to -771, -942 to +161 relative to the transcription start site) were amplified by PCR using mouse genomic DNA. Afterwards, these fragments were individually inserted into the pGL3-basic plasmid. To estimate the firefly luciferase activity, the above plasmids together with the phRL-TK plasmid were individually co-transfected into Hep 1-6 cells or Hep G2 cells, using Lipofectamine 2000 (Invitrogen, Carlsbad, USA) following the manual. After incubation for 6 h, the cells were cultured with fresh medium supplied with vehicle, CDCA or GW4064, or GW7647 and collected 24 h later. Dual-luciferase assay kit (Promega, USA) was used to detect the luciferase activity by using a Fluoroskan Ascent FL (Thermo Scientific, USA). Firefly luciferase activity was normalized with renilla luciferase activity as internal control.

### EMSA

Nuclear extracts were prepared from GW4064 or GW7647-treated livers using the Active Motif Nuclear Extract Kit (Active Motif, CA, USA) and BCA protein assay kit (Beyotime, Jiangsu, China) was used to detect the protein concentrations of nuclear extracts. Labeled and unlabeled probes (Sangon, Shanghai, China) containing the binding site were synthesized (Table S1). The DNA binding activity of FXR or PPARα was determined by a chemiluminescent EMSA Kit (Beyotime, GS009) according to instruction with minor modification to liver samples pretreatment.

### ChIP

GW4064-treated and GW7647-treated cells or livers were subjected to ChIP assays according to the protocol (Beyotime, P2078). Then the samples were immunoprecipitated with anti-FXR (Santa Cruz, sc-25039X), anti-PPARα antibody (Santa Cruz, sc-398394), anti-NCOR (Abcam, ab3482), anti-NCOR2 (Abcam, ab24551) and mouse IgG antibody (Beyotime, A7028) or recombinant rabbit IgG antibody (Abcam, ab172730) acting as a negative control. The captured chromatin was first eluted, and then uncross-linked, finally the DNA was recycled. Using the primer pairs (Table S1) spanning the specific promoter region, the captured DNA by ChIP was subjected to PCR amplification.

### Plasma transaminase levels and histological analysis

The ALT and AST levels in the plasma were measured with assay kits purchased from Nanjing Jiancheng (C009-2, C010-2). The livers were soaked in 4% formaldehyde for 1 day, and subsequently embedded in paraffin for histologic assessment. The prepared slices were further deparaffinized and stained with hematoxylin-eosin staining or Sirius red staining.

### Immunofluorescence staining

For histological analysis, the collected mouse livers were snap-frozen in OCT for preparation of frozen sections. Formalin-fixed paraffin-embedded tissue sections must be deparaffinized and subjected to antigen retrieval for satisfactory immunostaining. For immunofluorescence staining, the liver sections (6 μm) were blocked in PBS containing 10% goat serum and 1% Triton X-100, then the slices were incubated with primary antibodies, namely, anti-CK19 (Servicebio, GB12197), anti-GS (Santa Cruz, sc-74430), anti-GFP (Proteintech, 50430-AP or Santa Cruz, sc-9996), anti-BrdU (Servicebio, GB12051), anti-HNF4α (Ab41898) and anti-β-actin (Proteintech, 20536-1-AP). After being incubated with fluorophore-conjugated secondary antibodies (Invitrogen, A-11034, A-21424), slices were counter-stained with DAPI (Invitrogen, Ab104139). Finally, the results were observed under confocal microscope (LSM710, Carl Zeiss Microscopy GmbH or Nicol Laser Confocal Microscope).

### RNAscope^®^ *In situ* hybridization

Fluorescence ISH of *Lgr5*, *Gs*, *Pck1* and *Hnf4α* was carried out using the RNAscope^®^ ISH Assay (ACD, 323100), following the manufacturer's instructions. Concisely, prepared cryosections were fixed in formaldehyde for 15 min at 4 °C, dehydrated, and pre-treated in hydrogen peroxide for 10 min, followed by 0.5 h digestion in protease III. Prepared formalin-fixed, paraffin-embedded liver sections were baked, deparaffinized and then pre-treated in hydrogen peroxide, followed by target retrieval and protease digestion. Subsequently, slices were pre-amplified and amplified in terms of the directions. The resultant sections were counterstained using mounting medium with DAPI. The results were observed under laser confocal microscope (LSM710, Carl Zeiss Microscopy GmbH or Nicol Laser Confocal Microscope) with fixed parameters. Signal intensity was adjusted in each channel in term of their histograms. The obtained parameters after adjustment were used for the whole batch of pictures. The probe information was listed in Table S2.

### Software-Intensity measurement

Image Pro Plus (Image J), as an analysis program, was used to analyze and quantify data from photomicrographs. In this study, the analyses were performed as follows: Integrated optical density was used to quantify the intensity of probes binding to the structures 29. At least three mice per group and at least three confocal images were used for each mouse were analyzed.

### Statistical analysis

All data from experiments were presented as the mean ± SD. Statistical significance differences between groups were determined by Student's t-test or ANOVA. Statistical significance was set at *P*<0.05 (*), *P*<0.01 (**), *P*<0.001 (***). In all cases, data from at least 3 independent samples and 3 independent experiments were used.

## Results

### FXR activation induces *Lgr5* expression

The location of *Lgr5*^+^ cells in the adult liver was recently reported 30-32. In this study, we reproduced these results by PCR, WB, IF, RNAscope^®^ ISH and lineage tracing. As shown in Figure S1A-H, *Lgr5* was expressed in normal liver and *Lgr5*^+^ hepatocytes were mainly located around the perivenous (PV) area.

**Figure 1:**
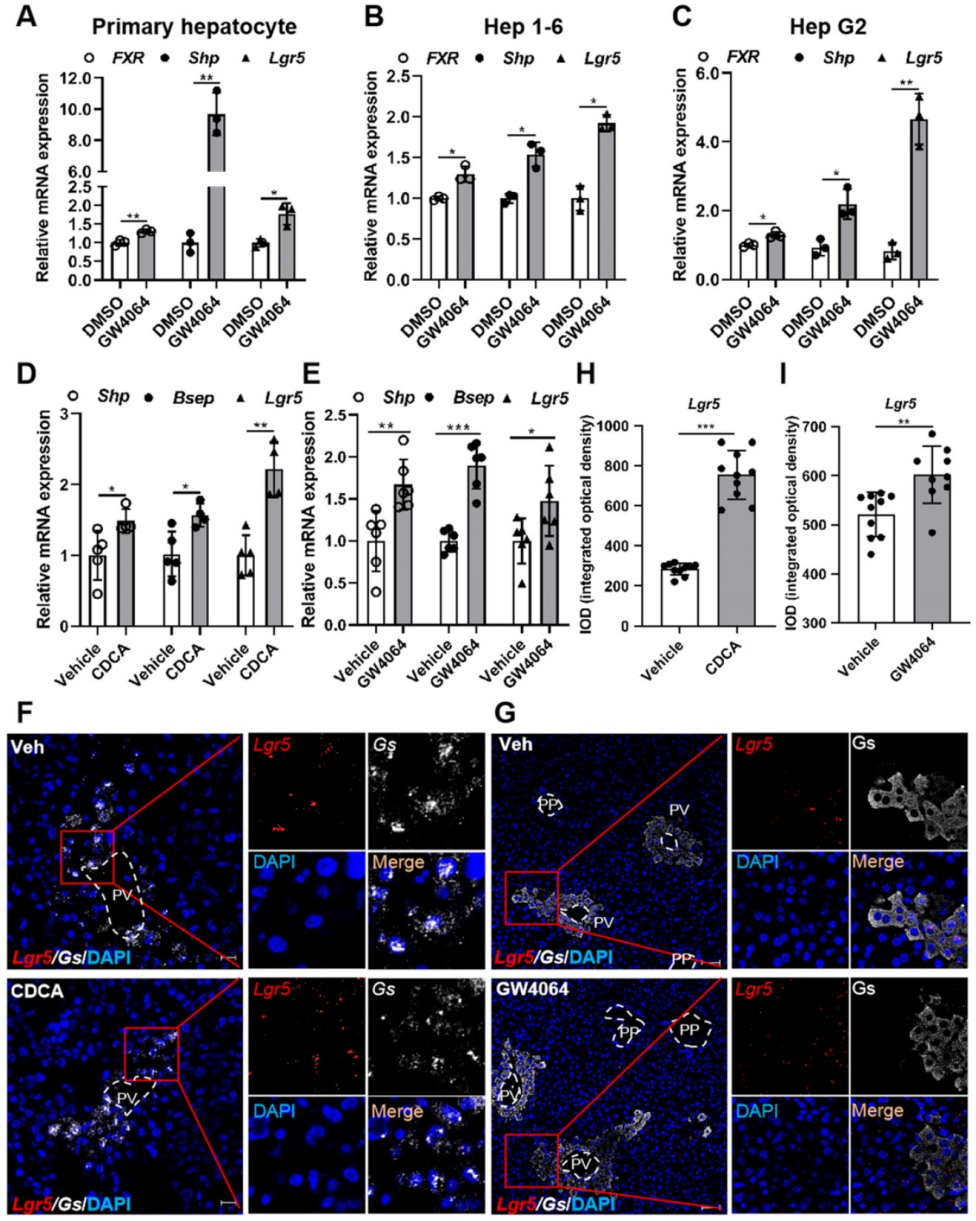
** Pharmacological activation of FXR induces *Lgr5* expression. (A-C)** QRT-PCR analysis of *FXR*, *Shp* and *Lgr5* expression in primary hepatocytes, Hep 1-6 cells and Hep G2 cells treated with GW4064 (10 µM) or DMSO for 24 h. **(D)** Mice were orally treated with either vehicle or CDCA (20 mg/kg) once a day for 7 days. Then, hepatic expression levels of *Lgr5*, *Shp* and *Bsep* were determined by QRT-PCR analysis. n = 5 per group. **(E)** Mice were orally treated with either vehicle or GW4064 (50 mg/kg) 2 days of twice-daily routines in a row. Then, hepatic expression levels of *Lgr5*, *Shp* and *Bsep* were determined by QRT-PCR analysis. n = 6 per group. **(F)** Representative images from RNAscope^®^ ISH assays for *Lgr5* and *Gs* on mouse liver cryosections treated with vehicle or CDCA. Red signal represents* Lgr5*, white represents *Gs*, blue shows DAPI. Scale bars, 20 µm. **(G)** ISH for *Lgr5* combined with IF for Gs was performed on mouse liver paraffin sections treated with vehicle or GW4064. Red signal represents *Lgr5*, white represents Gs, blue shows DAPI. Scale bars, 50 µm. **(H-I)** Quantification of the IOD for (F-G) was presented in the corresponding diagram. Gene expression level of the samples normalized to internal control and the average expression of interest genes in vehicle group was set as 1. Data were expressed as means ± SD, *p < 0.05, **p < 0.01 and ***p < 0.001 were determined by the two-tailed Student's t-test.

To determine whether FXR regulates *Lgr5* expression in liver, we treated primary hepatocytes, Hep 1-6 cells, and Hep G2 cells with FXR agonist GW4064. Results showed that GW4064 induced *FXR* expression, and small heterodimer partner (*Shp*) was up-regulated, resulting in FXR activation, thus increasing *Lgr5* expression (Figure 1A-C). These results indicate that FXR regulates *Lgr5* expression *in vitro*. Additionally, QRT-PCR and WB results indicated that the regulation was dose dependent in WT hepatocytes and was abolished by FXR^-/-^ mouse cells (Figure S2A-D).

To further confirm the results *in vivo*, FXR agonist CDCA or GW4064 was administrated intragastrically to WT mice. Up-regulation of *Shp* and bile salt export pump (*Bsep*) indicated the activation of FXR, which in turn strongly promoted *Lgr5* expression (Figure 1D-E). RNAscope^®^ ISH confirmed CDCA and GW4064 treatment resulted in increased *Lgr5* expression (Figure 1F-I). Thus, we conclude that FXR activation induces *Lgr5* expression *in vitro* and *in vivo*.

### PPARα activation suppresses *Lgr5* expression

**Figure 2:**
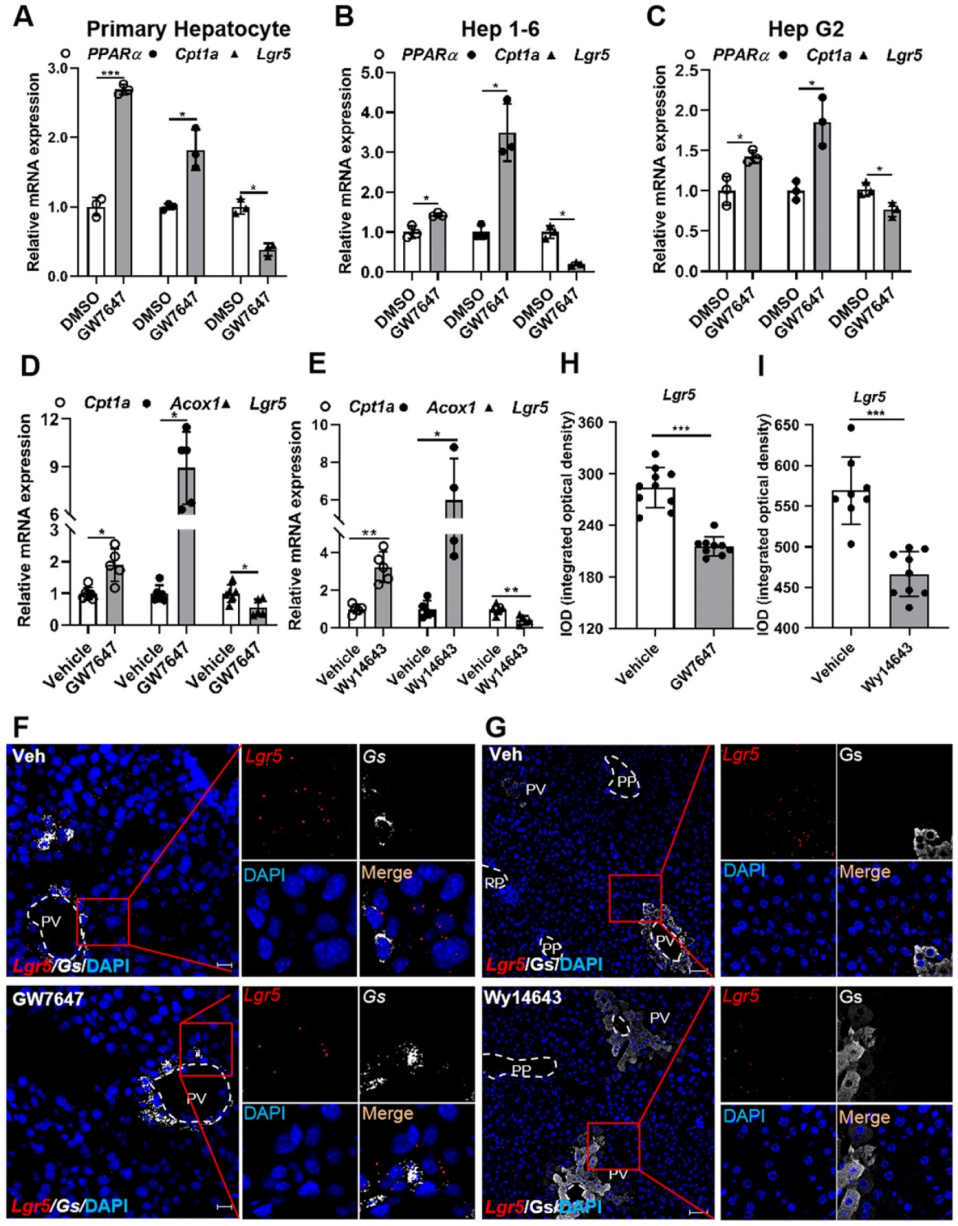
** Pharmacological activation of PPARα suppresses *Lgr5* expression. (A-C)** QRT-PCR analysis of *PPARα*, *Cpt1a* and *Lgr5* expression in primary hepatocytes, Hep 1-6 cells and Hep G2 cells treated with GW7647 (10 µM) or DMSO for 24 h. n = 3 per group. **(D)** Mice were orally treated with vehicle or GW7647 (5 mg/kg) 2 days of twice-daily routines in a row. Then, hepatic expression levels of *Lgr5*, *Cpt1a* and *Acox1* were determined by QRT-PCR analysis. n = 5 per group. **(E)** Mice were orally treated with vehicle or Wy14643 (100 mg/kg) 2 days of twice-daily routines in a row. Then, hepatic expression levels of *Lgr5*, *Cpt1a* and *Acox1* were determined by QRT-PCR analysis. n = 4-5 per group. **(F)** Representative images from RNAscope^®^ ISH assays for *Lgr5* and *Gs* on mouse liver cryosections treated with vehicle or GW7647. Red signal represents *Lgr5*, white represents *Gs*, blue shows DAPI. Scale bars, 20 µm. **(G)** ISH for *Lgr5* combined with IF for Gs was performed on mouse liver paraffin sections treated with vehicle or Wy14643. Red signal represents *Lgr5*, white represents *Gs*, blue shows DAPI. Scale bars, 50 µm. **(H-I)** Quantification of the IOD for (F-G) was presented in the corresponding diagram. Gene expression level of samples normalized to internal control and the average expression of genes in control group was set as 1. Data were expressed as means ± SD, *p < 0.05 and **p < 0.01 were determined by the two-tailed Student's t-test.

To examine whether PPARα regulates *Lgr5* expression in liver, primary hepatocytes, Hep 1-6 cells, or Hep G2 cells were treated with PPARα agonist GW7647. The results showed that GW7647 induced *PPARα* expression, and Carnitine palmitoyl transferase 1a (*Cpt1a*) was significantly up-regulated, thus activating PPARα, eventually decreasing *Lgr5* expression (Figure 2A-C). Additionally, QRT-PCR and WB results illustrated that different doses of GW7647 induces down-regulation of *Lgr5* expression in hepatocytes from WT mice and was abolished in PPARα KO mouse cells (Figure S2E-H). Similarly, PPARα activation by GW7647 reduced *Lgr5* expression in Huh 7 cells, and this reduction effect was abolished by PPARα inhibitor MK886 (Figure S3A). Besides, Wy14643, another ligand of PPARα, decreased the expression of *Lgr5* in primary hepatocytes (Figure S3B). These results indicate that PPARα activation suppresses *Lgr5* expression *in vitro*.

To further verify the results *in vivo*, mice were treated with the PPARα agonist GW7647 or Wy14643. The significantly increased expression of *Cpt1a* and Acyl-CoA oxidase 1 (*Acox1*) demonstrated the activation of PPARα (Figure 2D-E). GW7647 and Wy14643 drastically decreased *Lgr5* expression, and these results were further confirmed by RNAscope^®^ assays (Figure 2F-I). To determine whether PPARα specifically regulated *Lgr5* transcription, we treated WT and PPARα^-/-^ mice with GW7647 or vehicle. QRT-PCR results revealed that PPARα agonist notably suppressed the expression of *Lgr5* in WT, but this suppression effect was abolished in PPARα^-/-^ mice (Figure S3C-D). RNAscope^®^ ISH further confirmed these results (Figure S3E). Quantification of the signals was presented in Figure S3F. Based on above results, we conclude that PPARα activation reduces *Lgr5* expression *in vitro* and *in vivo*.

### Nuclear receptor FXR and PPARα regulate *Lgr5* transcriptional activation by binding to direct repeat (DR2) site on *Lgr5* promoter

**Figure 3:**
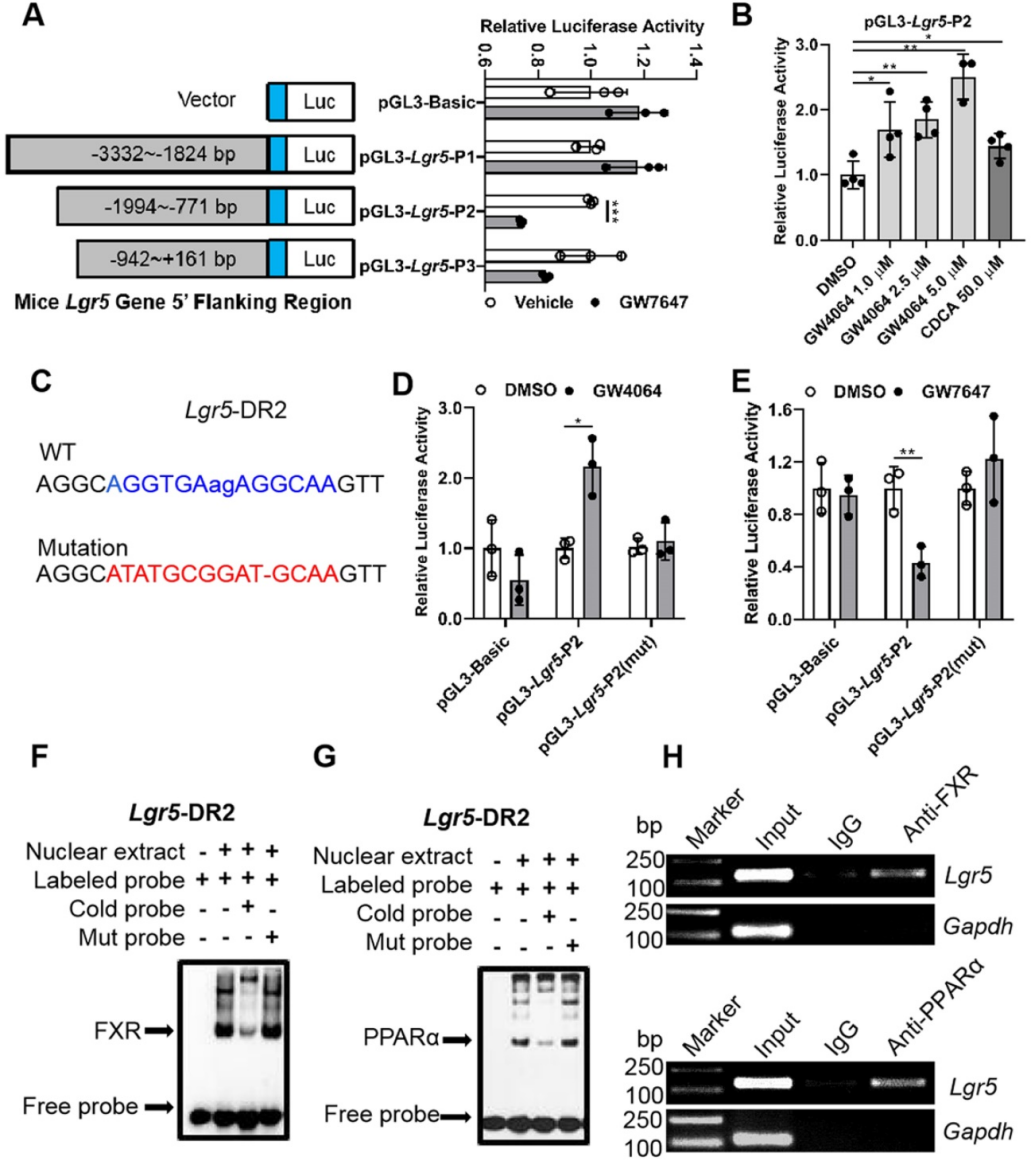
** Promoter of *Lgr5* contains putative FXR and PPARα binding site. (A)** Amplification and activity detection of different fragments of *Lgr5* promoter (position -3332 to -1824, -1994 to -771, -942 to +161 relative to the transcription start site). These fragments were inserted into the pGL3-Basic vector and transfected into Hep G2 cells with PPARα, RXR expression plasmids and pRL-TK using lipofectamine 2000. PPARα ligand GW7647 was added to cells for 24 h before the reporter assay. **(B)** Relative luciferase activity of the *Lgr5*-promoter reporter constructs when co-transfected into Hep G2 cells with FXR and RXRα expression plasmids and exposed to the indicated ligands. **(C)** Sequences of the WT and mutant *Lgr5*-DR2 sites. DR, direct repeat. 2, refers to the classical binding site separated by 2 bases (“ag” in lower case and highlighted in blue). “-” means base deletion. The blue DR2 nucleotides that were altered to form the mutant construct are marked red including “-”. **(D-E)** *Lgr5*-promoter or *Lgr5*-mutation plasmid was co-transfected into Hep 1-6 cells with pRL-TK. After incubation for 6 h, the cells were treated with DMSO, GW4064 or GW7647 for 24 h, respectively, and samples were analyzed by dual-luciferase assays. n = 3 per group. The ratio of firefly luciferase activity to renilla luciferase activity in control group was set to 1. **(F-G)** EMSA was performed to investigate the binding of liver nuclear proteins to the DR2. The position of the shifted NR/DR2 complex and free probes are indicated by arrows. **(H)** ChIP analysis showed that FXR or PPARα interacted with DR2 elements in Hep 1-6 cells treated with GW4064 or GW7647. Data were expressed as means ± SD, n = 3 independent experiments containing 3 replicates, *p < 0.05, **p < 0.01 and ***p < 0.001 were determined by the two-tailed Student's t-test or one-way ANOVA.

In general, nuclear receptors activate or repress target gene expression by directly binding to DNA response elements. In order to reveal the impact of both FXR and PPARα on *Lgr5*, luciferase assay kit was used to screen the most possible binding site. Results indicated that GW4064 promoted FXR binding to peaks corresponding to *Lgr5* to increase its transcription. On the contrary, GW7647 treatment suppressed *Lgr5* transcriptional activity (Figure 3A-B). Next, we carried out site-directed mutagenesis of the binding element, which abolished the reporter expression regulated by FXR and PPARα activation (Figure 3C-E). These results indicate that *Lgr5* may be directly regulated by FXR and PPARα via binding to DR2 site.

EMSA results showed that the interaction between labeled probe and the nuclear extracts of mouse liver samples treated with GW4064 or GW7647 exhibited a DNA-protein band shift with expected mobility rate. Such an interaction was competitively inhibited by adding excessive unlabeled probes, rather than by adding mutation probes (Figure 3F-G). Subsequently, the specific precipitated elements were obtained by ChIP, indicating that FXR and PPARα could specifically interact with DR2 site, which was consistent with the above results (Figure 3H). Additionally, we combined different doses of the ligands to activate FXR and PPARα simultaneously, and the upregulation of* Lgr5* suggested a dominant effect of FXR on *Lgr5* regulation under the current conditions (Figure S4A-B). In addition, NCOR and NCOR2 are the most studied PPARα corepressors 33-35. ChIP-PCR results showed that GW7647 increased NCOR and NCOR2 corepressors binding to the same DR2 element (Figure S4C), this might be why PPARα activation inhibits* Lgr5* expression. Collectively, the above results suggest that FXR and PPARα mutually competed for binding to the same promoter element of *Lgr5*, but they drive opposite transcriptional outputs.

### Activation of FXR or PPARα alters *Lgr5* expression and *Lgr5*^+^ cell proliferation after DDC diets

We further investigated effects of FXR and PPARα on *Lgr5*^+^ cells-supported recovery from DDC-induced liver damage. Hematoxylin-eosin staining results indicated that ductular reaction and inflammatory infiltration were observed in all the mouse livers after 1 wk DDC treatment (Figure S5A). We also observed that FXR and PPARα activation rescued liver injury by attenuating plasma AST and ALT levels in WT mice fed with DDC diet (Figure S5B-C). For morphometric analysis, the bile duct was marked by CK19 and normalized to the area of portal veins showing significantly increased CK19-positive area/portal field in DDC-fed mice, which was accompanied by the development of portal-portal septa and the increasing *TNFα* mRNA levels (Figure S5D-H). In summary, DDC-fed WT mice exhibited pronounced symptoms of liver injury such as bile duct hyperplasia and focal necrosis, and these symptoms were relieved by FXR and PPARα activation.

**Figure 4:**
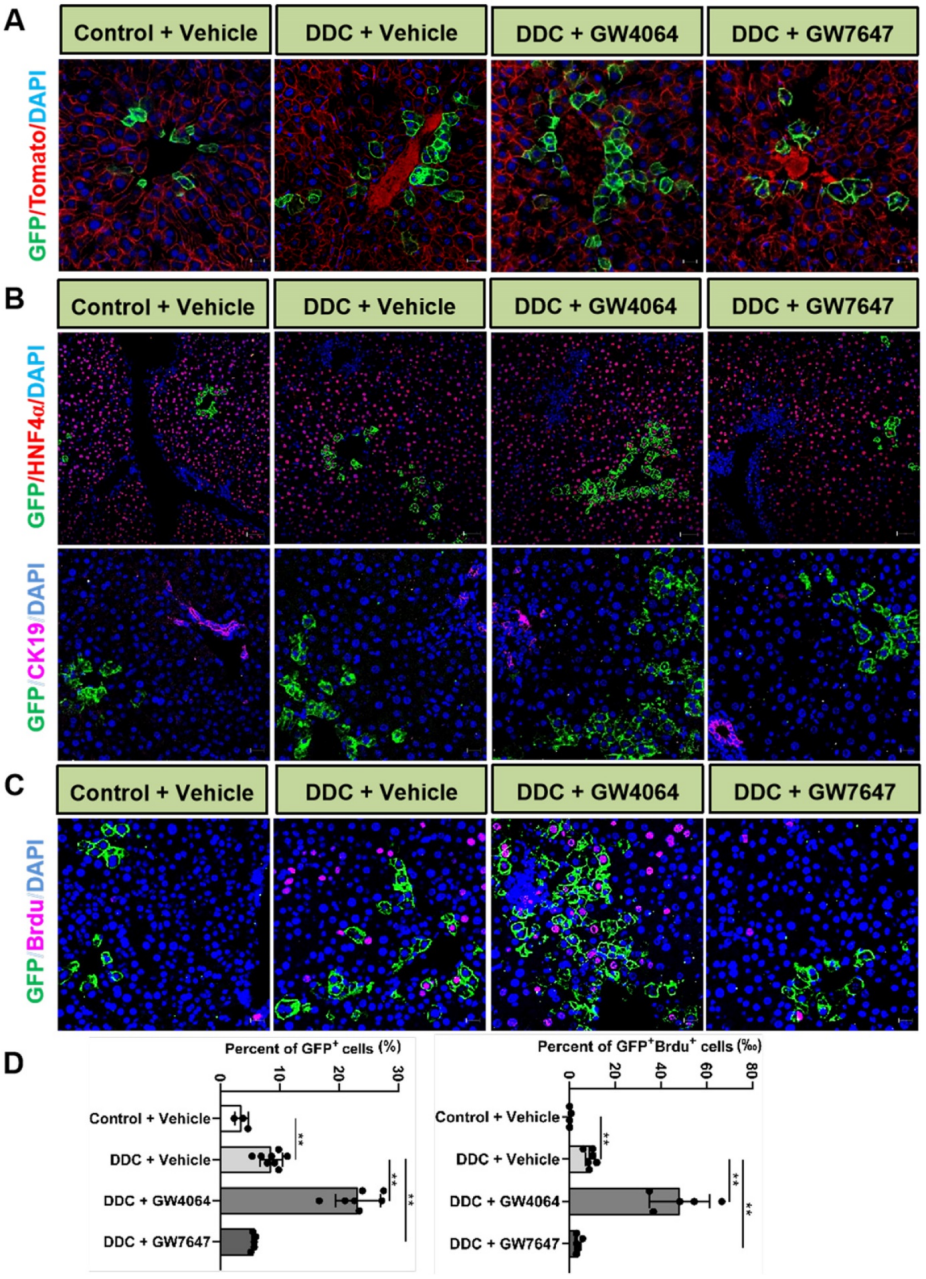
** Activation of FXR or PPARα alters *Lgr5* expression and *Lgr5*^+^ cells propagation after DDC diets**. Liver sections of *Lgr5*-Cre^ERT2^; Rosa26-mTmG mice treated with tamoxifen (100 mg/kg) were analyzed. Mice were fed with DDC diet for 1 wk and treated with GW4064 or GW7647. **(A)** Representative images from fixed frozen liver sections showing lineage tracking of *Lgr5* cells upon DDC damage. GFP (green) fluorescence shows *Lgr5*^+^ hepatocytes and the offspring. Tomato fluorescence shows *Lgr5*^-^ hepatocytes. Scale bars, 20 µm. **(B)** Representative images showing tracking of *Lgr5* cells upon DDC damage. Co-staining results of GFP (Green) with HNF4α (Red, scale bars, 50 µm) or CK19 (Violet, scale bars, 20 µm). **(C)** GFP and BrdU double staining livers after DDC injury. Blue (DAPI) shows nuclei, violet (BrdU) marks the proliferating cells, green represents *Lgr5^+^ cells and their offspring cells*. Scale bar represents 20 µm. **(D)** Graphs showing percentages of GFP^+^ cell and GFP^+^ BrdU^+^ cell in the indicated groups. Data were expressed as means ± SD, n = 3 independent experiments containing 4 replicates, **p < 0.01 were determined by one-way ANOVA.

To further reveal the effects of FXR and PPARα activation on the cell fate of *Lgr5*, *Lgr5*-Cre^ERT2^; Rosa26-mTmG heterozygous mice were treated with DDC diet along with GW4064 or GW7647. We found that FXR activation promoted the expansion of *Lgr5*^+^ cells and their progeny in the PV region, while PPARα inhibited the expansion of *Lgr5*^+^ cells and their progeny (Figure 4A). Co-staining results of GFP with HNF4α or CK19 antibody revealed that GFP^+^ cells existed in the hepatocytes rather than in bile duct cells (Figure 4B). In addition, few GFP^+^ cells were labeled with BrdU in the control group. Upon DDC injury, BrdU incorporation was enhanced with an increasing number of GFP^+^ cells, and FXR activation further increased ratio of GFP^+^BrdU^+^ cells from 9.17‰ to 44.19‰, and the GFP^+^ cells migrated distally, away from PV to PP, whereas PPARα activation maintained the quiescence of *Lgr5*^+^ cells and their offspring (Figure 4C-D). The number of EdU positive cells at 24 h after EdU injection was significantly more than that of at the 4 h after EdU injection in DDC diet mice liver samples (Figure S6A-B). As illustrated in Figure S6C, nearly all EdU-labeled cells co-localized with BrdU-positive cells, these data suggest that EdU and BrdU staining methods detected DNA synthesis with equal efficiency. Based on these results, we conclude that EdU/BrdU^+^GFP^+^ cells are the result of daughter cell proliferation.

**Figure 5:**
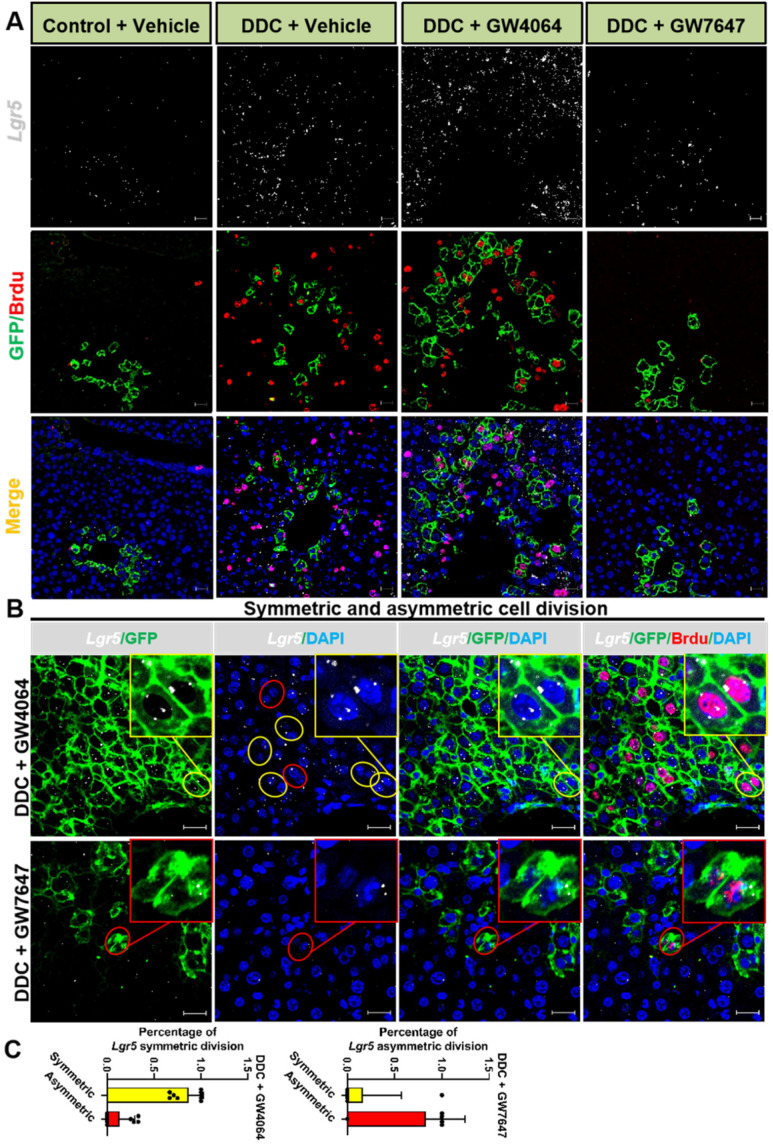
***Lgr5*^+^ cells predominately undergo symmetric division while FXR activation but turn on asymmetric division while PPARα activation. (A)** ISH for *Lgr5* combined with IF for GFP and BrdU was performed to confirm *Lgr5* expression and *Lgr5*^+^ stem cell proliferation. **(B)** Yellow circle depicts the symmetric division, both of the daughter cells with *Lgr5* expression, and red circle depicts the asymmetric division, the stem cell with *Lgr5* expression (white) and another daughter cell without *Lgr5* expression. Scale bar represents 20 µm. **(C)** Quantification of* Lgr5* symmetric or asymmetric division in GFP^+^ liver stem cell maintained in livers. n = 3 per group. Data were expressed as means ± SD.

Previous study has reported that although multiple adult stem cells are likely to divide asymmetrically under homeostasis, they still have the ability to divide symmetrically to replenish the stem-cell pool whose exhaustion was caused by injury or disease 36. Therefore, we examined whether FXR and PPARα had influence on liver injury repair via cell division of *Lgr5*^+^ cells. The co-staining results of *Lgr5* probe, GFP antibody, and BrdU antibody indicated that FXR activation promoted *Lgr5*^+^ cells proliferation mainly by symmetric cell divisions to generate more stem cells with *Lgr5* expression (Figure 5A-C and Figure S7A). On the contrary, PPARα activation preferentially inhibited *Lgr5* cells proliferation by asymmetric cell division to maintain self-renewal or static state (Figure 5B-C and Figure S7A). Furthermore, ISH for *Lgr5* combined with IF for BrdU and β-actin multiple fluorochrome labeled antibody hybridization were performed to test *Lgr5* expression and *Lgr5*^+^ stem cell proliferation. DDC-injured GW4064 treated mouse liver exhibited symmetric cell division with both of the daughter cells expressing *Lgr5*, while GW7647 treated group showed asymmetric cell division with one stem cell expressing *Lgr5* and one differentiated cell without *Lgr5* expression (Figure S7B-C).

Taken together, FXR and PPARα oppositely regulate cell fate of *Lgr5*^+^ and their offspring under hepatic physiological and pathological conditions.

## Discussion

Although* Lgr5*^+^ cells have been reported to be beneficial for liver damage repair 5, the expression of *Lgr5* in the liver has been controversial. Some research indicates that *Lgr5* only appears near bile ducts after liver damage 5, but other research reveals that *Lgr5* is expressed in normal adult liver 31, 32, 37. Unfortunately, the previous studies failed to accurately mimic the expression pattern of endogenous *Lgr5* due to the single detection method. In this study, combining WB, IF, RNAscope^®^ ISH and lineage tracing, we reproduced that *Lgr5* was expressed in normal liver and *Lgr5*^+^ cells were located in the PV area. Previous research indicates that β-catenin signal is activated in all of the PV hepatocytes 38. Since *Lgr5* was a downstream target gene of Wnt/β-catenin pathway, our results that *Lgr5* was located around PV area were consistent with those from the point of view of metabolic zoning and anatomical localization 39, 40.

Metabolic nuclear receptors have been found to regulate the fate of *Lgr5*^+^ stem cells in the intestinal tract through Wnt pathway and diet 11, 41. However, whether nuclear receptors can regulate the fate of *Lgr5* hepatocytes during liver regeneration and repair has not been documented. FXR and PPARα are essential regulators of metabolic responses and highly expressed in hepatocytes 42, 43. FXR is activated by endogenous BA synthesized from cholesterol in the PV hepatocytes. PPARα is up-regulated at PP area with high oxygen concentration, thus intrinsically raising key biological and metabolic activity 44, 45. In this study, we found that activation of FXR promoted *Lgr5* expression and activation of PPARα inhibited *Lgr5* expression both *in vitro* and *in vivo*. Previous research has also indicated that FXR and PPARα coordinate autophagy by directly competing for occupancy at the same site on autophagy-related gene promoter 20. Consistent with their results, our data showed that FXR and PPARα exhibited opposite regulatory effects on *Lgr5* transcription by directly binding to the shared promoter element (DR2). Moreover, FXR and PPARα function coordinately to control pivotal nutrient pathways and to maintain liver energy balance and that may alter liver stem cell fate 46. After a meal, hepatic FXR is activated by the BA that return to the liver 46 and it may affect *Lgr5* stem cell behavior. Meanwhile, dietary treatment with bile acid cholic acid reportedly inhibited primary PPARα targets, such as *Acox1*, which may downregulate fatty acid oxidation levels 46 and result in hindering *Lgr5^+^* stem cell expansion. These effects are consistent with their expected roles as mediators of the feeding and fasted responses and are in agreement with our results that a dominant effect on *Lgr5* regulation by *FXR* under physiological conditions. Previous study has reported that high expression of *Lgr5* is observed in chronic liver disease, and that *Lgr5*^+^ liver cells probably contribute to liver function recovery 6. Notably, the dynamics of *Lgr5* expression post damage suggest that *Lgr5* should be expressed in the early stage of the injury initiation and it should be switched off once the tissue regeneration is finished. Based on these findings, it could be speculated that *Lgr5* might act as a switch for cell proliferation 47. Accordingly, this study proposed a local expansion manner of hepatocytes in liver homeostasis and regeneration. Lineage tracing results have shown that *Lgr5*^+^ cells in mice fed with normal diet maintained static state, that DDC diet triggered the transition of *Lgr5*^+^ cells from a resting state to a proliferating state, and that activation of FXR or PPARα altered *Lgr5^+^* liver stem cell propagation, thus alleviating liver injury after DDC diet. This result is in line with the previous conclusion that FXR and PPARα can promote liver regeneration and repair 48-50. Our data indicated that during liver injury repair, FXR activation drove the expansion of *Lgr5*^+^ cells around the PV region, and PPARα had opposite effects. On one hand, the co-regulation of *Lgr5* by FXR and PPARα promoted the rapid proliferation of *Lgr5*^+^ cells upon injury, on the other hand, co-regulation could prevent the excessive proliferation of *Lgr5*^+^ cells and their progeny, which might explain the mechanism of initiation and termination of liver regeneration to some degree. Thus, *Lgr5* could mark those cells that exhibited high plasticity and could freely switching between stem cells and differentiation cells. Additionally, upon DDC injury, GW4064 or GW7647-treated mice exhibited some *Lgr5*^-^/BrdU^+^ or GFP^-^/BrdU^+^ cells, these should also be taken into consideration. The liver with alternative stem cell niches exhibited strong adaptability to maintain a dynamic balance after chronic liver injury. And based on our previous research, specific FXR agonists given orally can activate intestinal FXR, which may regulate hepatocyte proliferation though FXR-FGF15-BA axis should also be taken into consideration 13. Beyond that, activation of intestinal FXR shaped the gut microbiota to activate TGR5/GLP-1 signaling to improve liver function 51. More investigation needs to carry out to show how intestine FXR activation contributes to the liver regeneration during chronic liver injury.

There are two types of cell division to ensure the self-renewal of mammalian stem cells in the process of proliferation. Asymmetrical cell division produces one stem cell and one differentiation cell to balance stem cell numbers for homeostasis maintenance, while symmetrical cell division generates two daughter stem cells to enlarge the stem cell pool for tissue regeneration 52, 53. Herein, we found that FXR promoted the symmetric cell division of *Lgr5*^+^ cells for liver regeneration, and PPARα tended to induce asymmetric division of *Lgr5*^+^ cells and their offspring for self-renewal or quiescence maintenance. The symmetrical division ability of stem cells enhanced tissue developmental plasticity and liver regeneration capacity, but over-proliferation might cause cancer, and PPARα activation could effectively resist cancer risk. Thus, we proposed that the switch between symmetric and asymmetric divisions of *Lgr5* stem cells was regulated by FXR and PPARα activation. This regulatory switch is closely associated with basic stem-cell biology, and has great significance to the clinical application of stem cells.

## Conclusions

In generally, our results reveal intersecting and complementary genomic circuits in which nuclear receptors induced or suppressed *Lgr5* expression. Our research also illustrates FXR and PPARα may be potential drug targets for liver diseases since they can regulate *Lgr5*^+^ cells fate. This study provides useful information for maintaining homeostasis of liver, and it will be of great significance for exploring the pathogenesis mechanism of human diseases and therapeutic strategies.

## Supplementary Material

Supplementary figures and tables.

## Abbreviations

*Acox1*: acyl-CoA oxidase1
BA: bile acid
*Bsep*: bile salt export pump
ChIP: Chromatin immunoprecipitation
Ck19: Cytokeratin 19
*Cpt1a*: Carnitine palmitoyl transferase 1a
DDC: diethyl1,4-dihydro-2,4,6-trimethyl-3,5-pyridinedicarboxylate
EMSA: electrophoretic mobility shift assay
*FXR*: farnesoid X receptor
GFP: green fluorescence
HCC: hepatocellular carcinoma
Hnf4α: hepatocyte nuclear factor 4α
IF: immunofluorescence
ISH: *in situ* hybridization
KO: knockout
*Lgr5*: leucine-rich repeat-containing G protein-coupled receptor 5
PP: periportal
*PPARα*: peroxisome proliferator-activated receptor α
PV: perivenous
*Shp*: small heterodimer partner
WT: wild-type
wk: week
