# Supplementary material for: Lgr5+ cell fate regulation by coordination of metabolic nuclear receptors during liver repair
Source: Theranostics. 2022 Aug 15;12(14):6130–42. doi: 10.7150/thno.74194 (PMC9475469; doi:10.7150/thno.74194)
Supplement: Supplementary file 1 — Supplementary figures and tables. [file thnov12p6130s1.pdf]

Supplemental Information

Supplemental Figure and Figure Legends

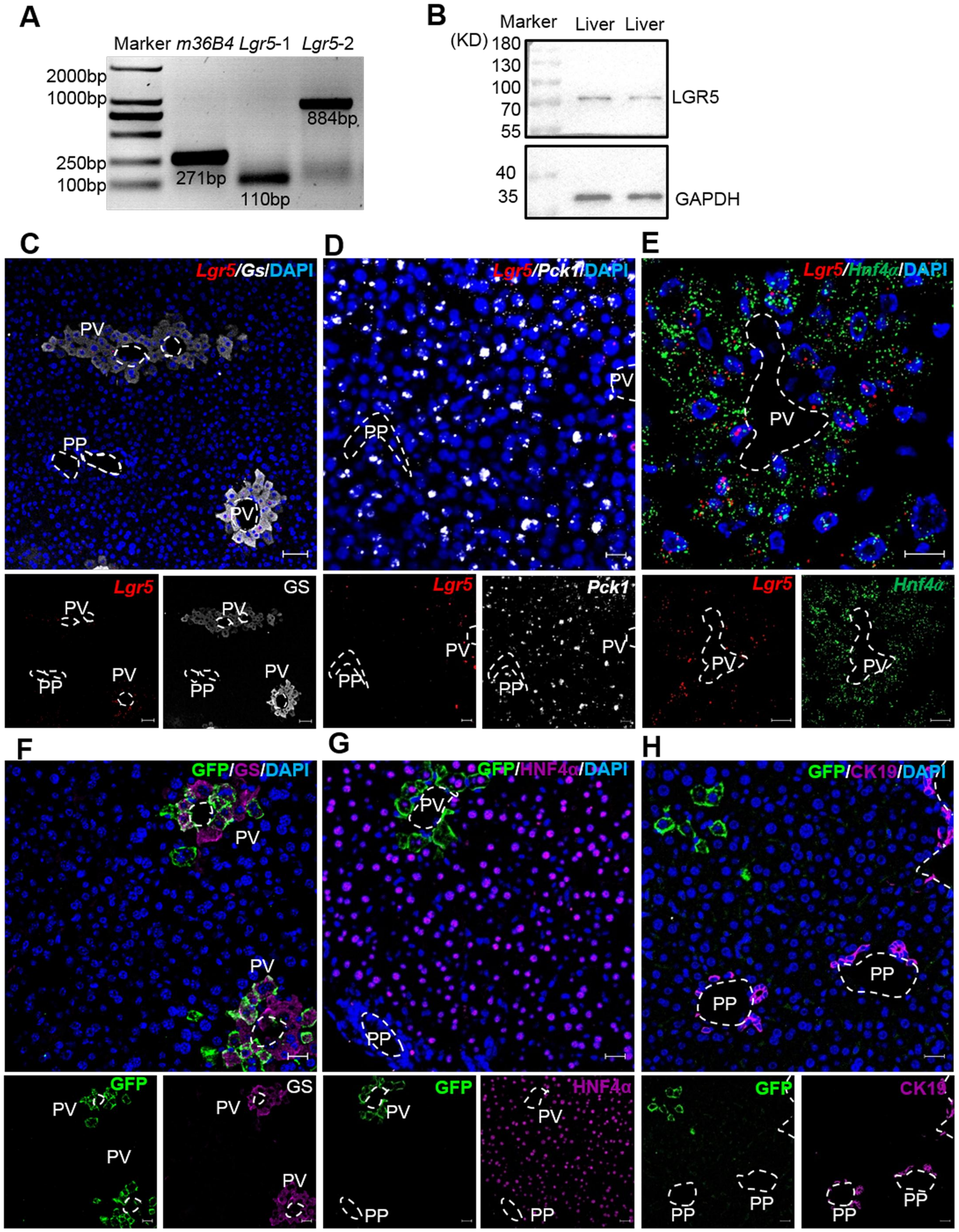

Figure S1 *Lgr5* is mainly expressed in hepatocytes adjacent to pericentral vein. (A) *Lgr5* expression was assessed in mouse livers by PCR. *Lgr5*-1 [1] and *Lgr5*-2 [2] represent *Lgr5* primers from two different literatures. (B) Western blot analysis of LGR5 in liver samples. (C-E) Representative images from RNAscope® assay for *Lgr5* mRNA in liver. Red represents *Lgr5*, white marks *Gs* hepatocytes (Scale bars, 50 μm) or *Pck1* hepatocytes in PV or PP zone, green shows *Hnf4α*, blue shows DAPI. Scale bars, 20 μm. (F-H) GFP was co-stained with GS or HNF4α or CK19 in the liver sections by IF. *Lgr5*$^{+}$ cells and their progeny were labeled by GFP. Scale bars, 20 μm.

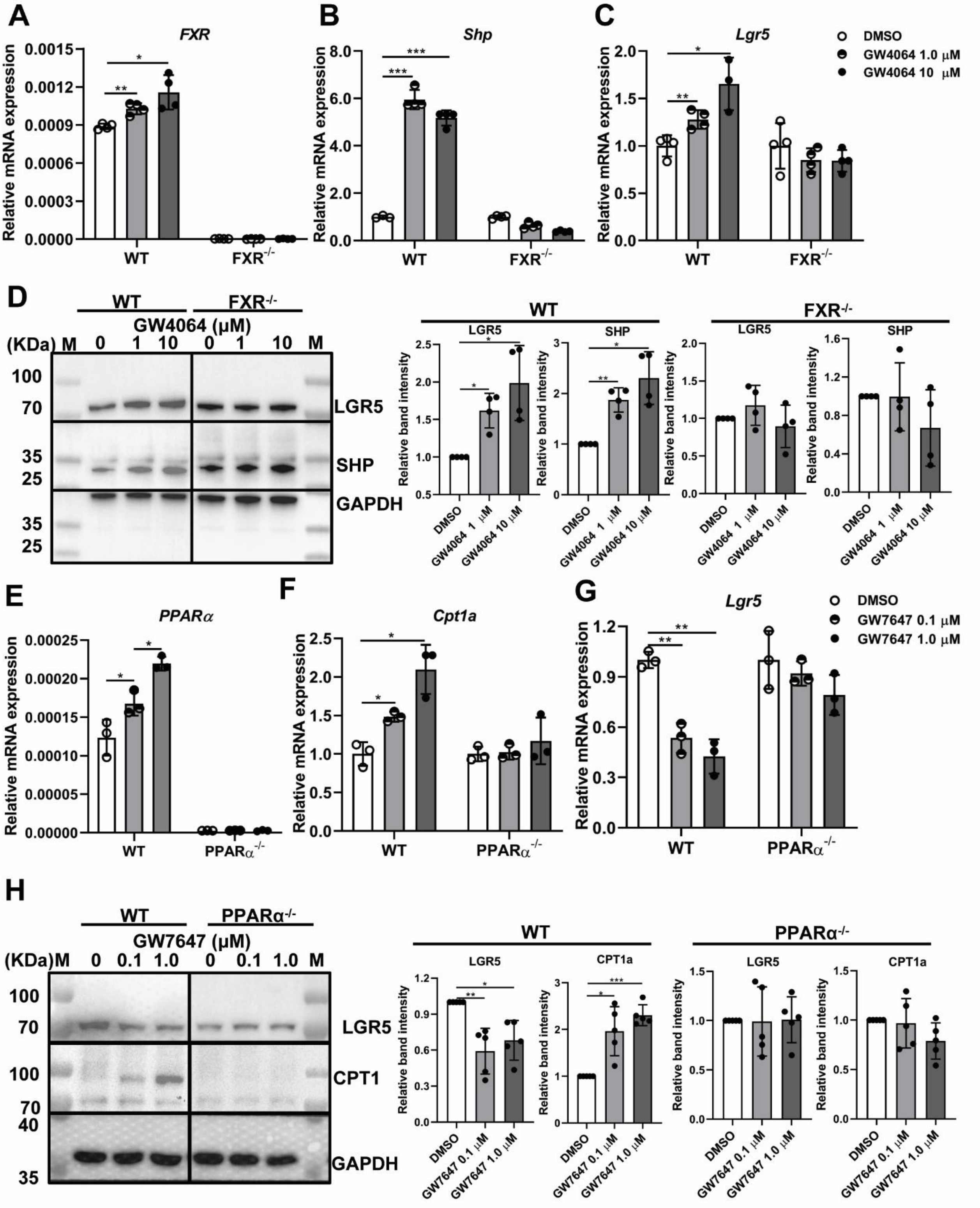

Figure S2 FXR and PPARα agonists affect *Lgr5* expression in mouse primary hepatocytes. (A-C) QRT-PCR analysis of *FXR*, *Shp* and *Lgr5* expression in primary hepatocytes treated with indicated concentration of GW4064. (D) Western blot analysis of

LGR5 and SHP in primary hepatocytes treated with GW4064. Result of GAPDH was obtained by membrane reuse of SHP. Protein levels of LGR5 and SHP were calculated by Image J. (E-G) QRT-PCR analysis of *PPARα, Cpt1a* and *Lgr5* expression in primary hepatocytes treated with indicated concentration of GW7647. (H) Western blot analysis of LGR5 and CPT1a in primary hepatocytes treated with indicated concentration of GW7647. Result of LGR5 was obtained by membrane reuse of CPT1a. Protein levels of LGR5 and CPT1a were calculated by Image J. The mRNA level of the interest gene was normalized to housekeeping gene *36B4* or *Gapdh* and the average expression of interest gene in vehicle group was set as 1. In order to reflect the knockout effects, QRT-PCR results showed the original expression levels of *FXR* and *PPARα*. Protein expression levels of the samples normalized to GAPDH and the expression of interest genes in vehicle group were set as 1. Data were expressed as means ± SD, *$p < 0.05$, **$p < 0.01$ and ***$p < 0.001$ were determined by one-way ANOVA.

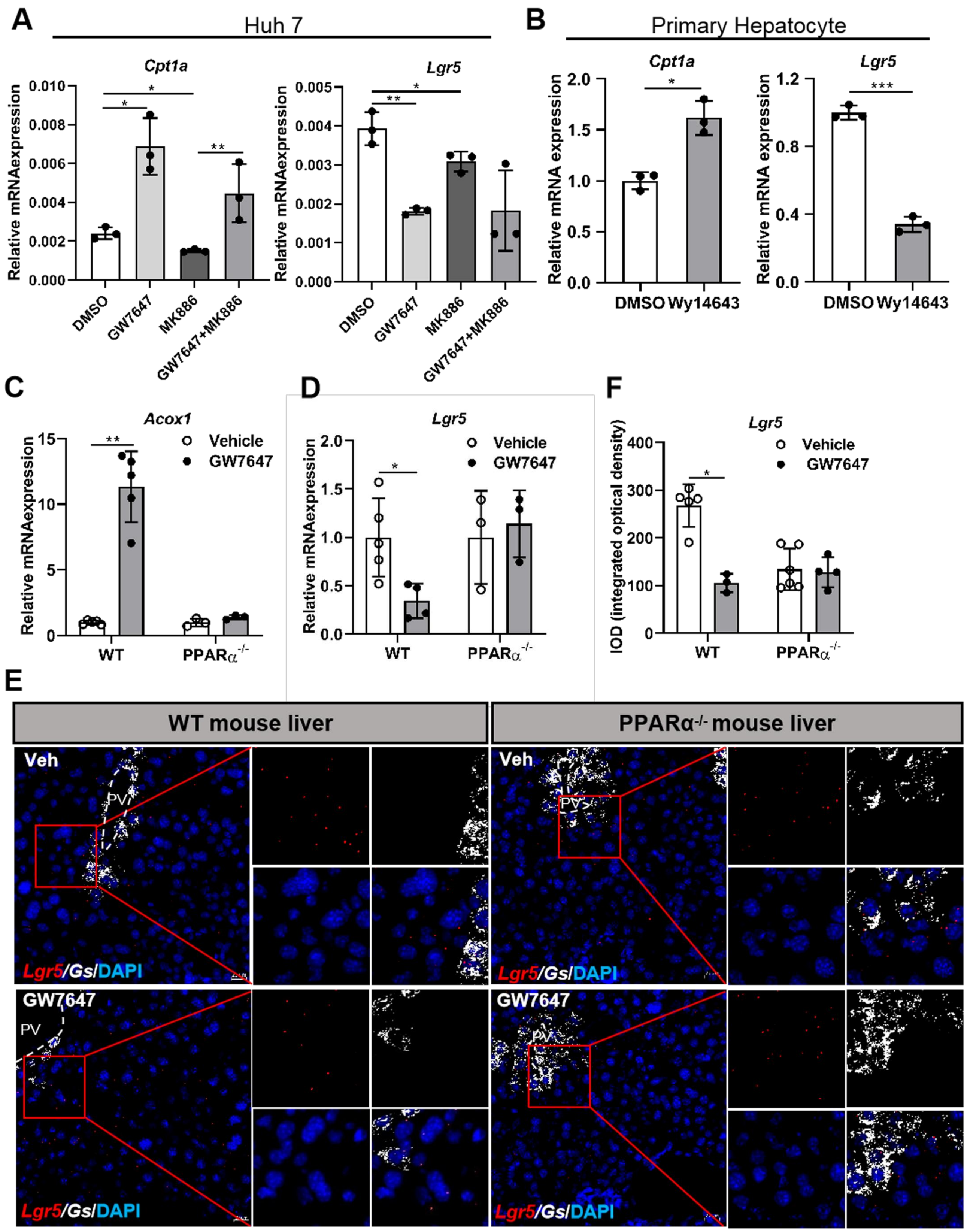

Figure S3 Activation of PPARα decreases *Lgr5* expression. (A) QRT-PCR analysis of *Cpt1a* and *Lgr5* expression in Huh 7 post-treatment with indicated chemical. (B) QRT-PCR

analysis of *Cpt1a* and *Lgr5* expression in primary hepatocytes treated with Wy14643. (C-F) WT and *PPARα*$^{-/-}$ mice were orally treated with vehicle or GW7647. (C-D) QRT-PCR analysis of *Acox1*, and *Lgr5* expression in mouse livers. (E) Representative images from RNAscope® assays for *Lgr5* on mouse liver section. Red represents *Lgr5*, white marks *Gs* and blue shows DAPI. Scale bars, 20 μm. (F) Quantification of the IOD was presented in the corresponding diagram. Data were expressed as means ± SD, *$p < 0.05$, **$p < 0.01$ and ***$p < 0.001$ were determined by the two-tailed Student's t-test or one-way ANOVA.

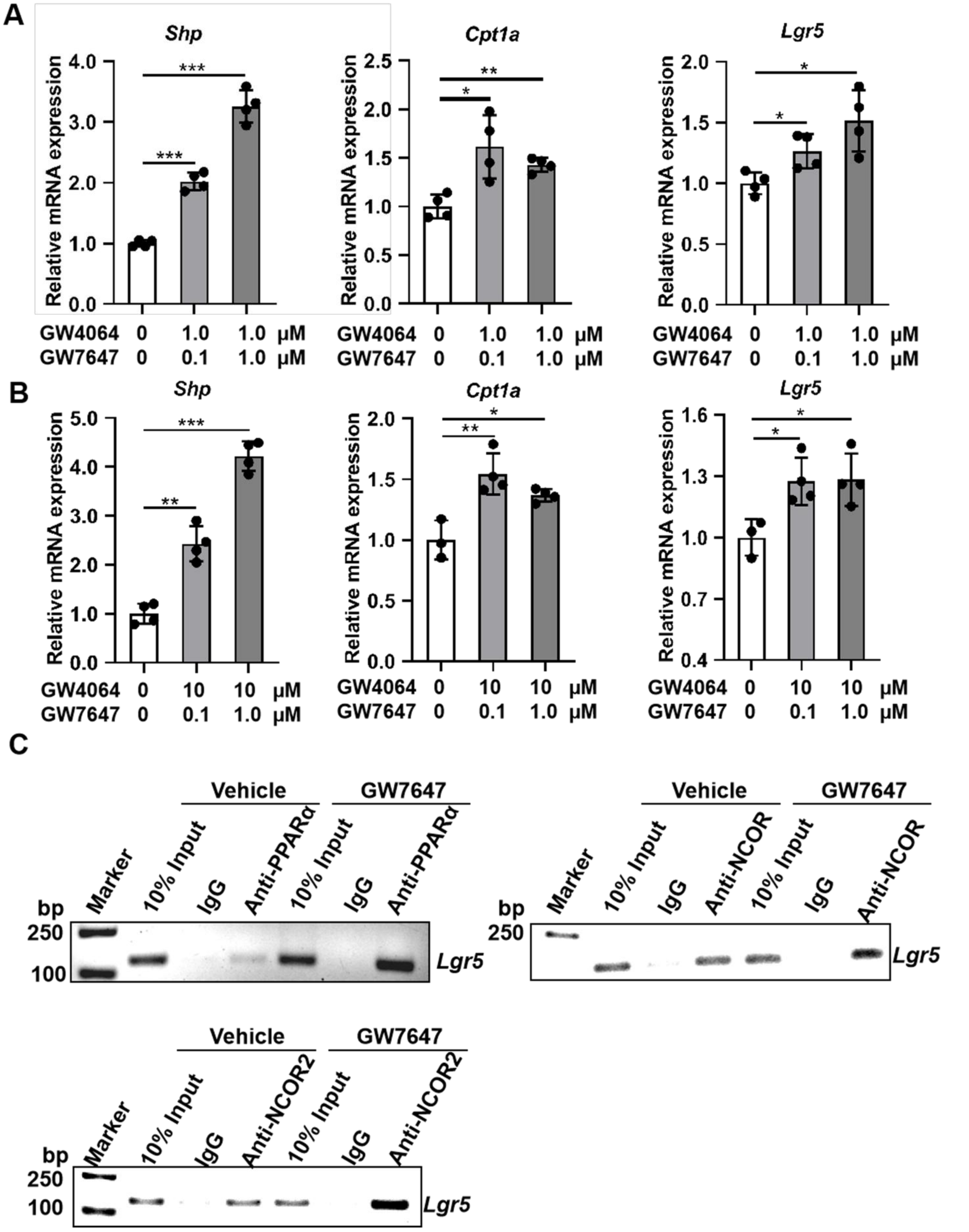

Figure S4 Activation of FXR and PPARα control *Lgr5* expression in mouse hepatocytes. (A-B) Primary hepatocytes were isolated from WT mice and then co-treated

with indicated concentration of GW4064 and GW7647 for 24 h. *Shp*, *Cpt1a* and *Lgr5* expression were detected by QRT-PCR. (C) WT mice were orally gavaged with vehicle or GW7647 twice a day for 2 days. Hepatic ChIP-PCR analysis with indicated antibodies (PPARα, NCOR and NCOR2) was used to determine recruitments of co-regulators by PPARα binding to DR2 in the promoter region of *Lgr5*. Data were expressed as means ± SD, n = 3 independent experiments containing 4 replicates, *$p < 0.05$, **$p < 0.01$ and ***$p < 0.001$ were determined by the two-tailed Student's t-test or one-way ANOVA.

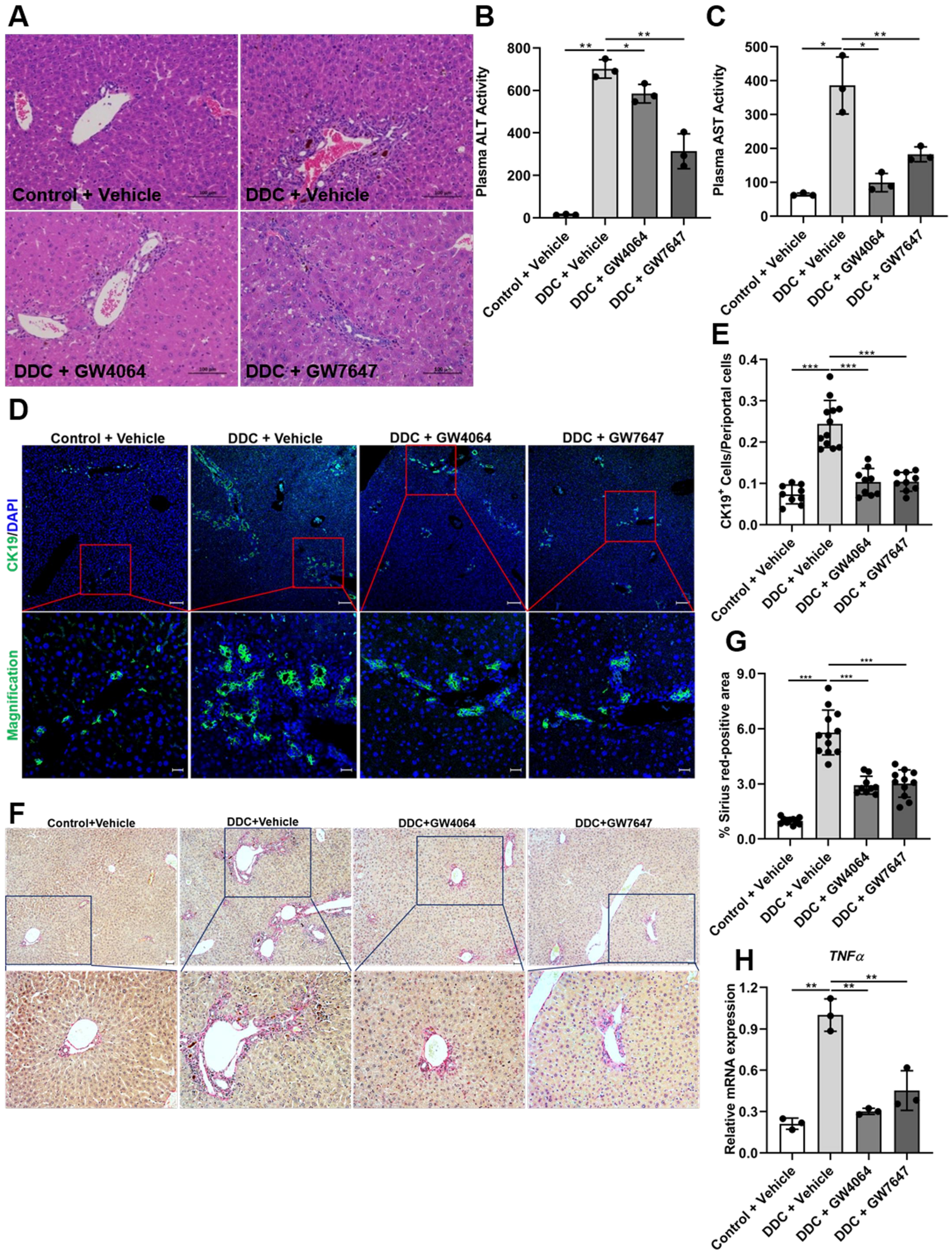
A
Control + Vehicle
DDC + Vehicle
DDC + GW4064
DDC + GW7647
B
Plasma ALT Activity
C
Plasma AST Activity
D
CK19/DAPI
Magnification
E
CK19+ Cells/Periportal cells
F
Control+Vehicle
DDC+Vehicle
DDC+GW4064
DDC+GW7647
G
% Sirius red-positive area
H
TNFα
Relative mRNA expression

Figure S5 Activation of FXR or PPARα alleviates liver injury. Mice were fed with DDC diet for 1 wk and were orally gavaged with vehicle, GW4064 or GW7647 twice every other day. (A) Histopathological analysis of representative mouse liver samples following Hematoxylin-eosin staining. Scale bars, 100 μm. (B-C) Serum ALT and AST levels were in the indicated groups. n = 3 mice per group. (D) The images were overlays of CK19 (Green) and DAPI (Blue) staining of the liver samples. Scale bars, 100 μm. (E) Graphs showing percentages of CK19-positive area/portal field in DDC-fed mice. (F) Sirius red staining in control liver and livers of mice fed 0.1% (w/w) DDC-supplemented diet for 1 wk with vehicle, GW4064 or GW7647 treatment. (G) Graphs showing percentages of Sirius red positive area in the indicated groups. (H) QRT-PCR analysis of *TNFα* mRNA expression in livers post-treatment with indicated chemical. Data were expressed as means ± SD, $^{*}p < 0.05$ and $^{**}p < 0.01$ were determined by one-way ANOVA.

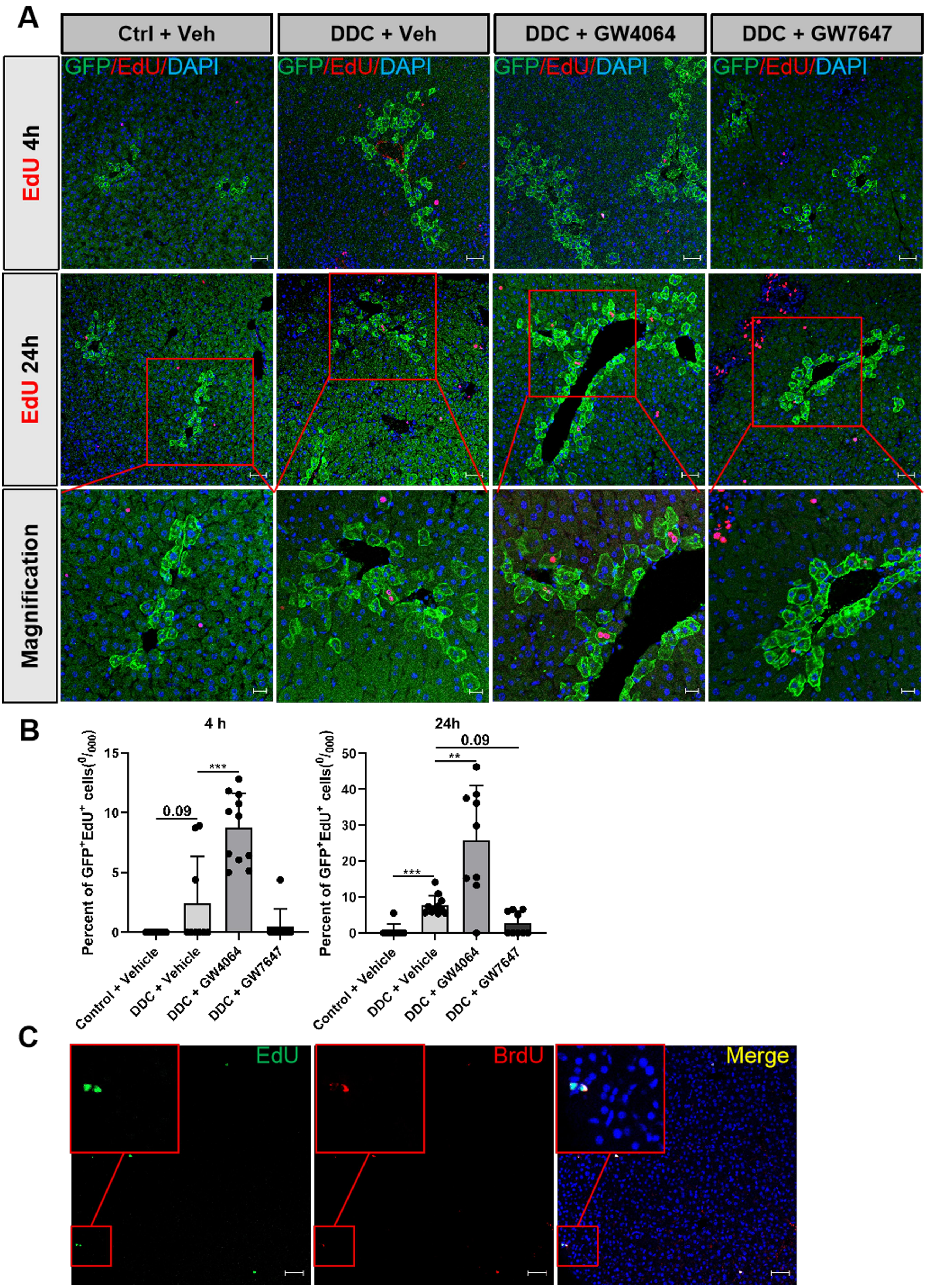
A
Ctrl + Veh
DDC + Veh
DDC + GW4064
DDC + GW7647
GFP/EdU/DAPI
EdU 4h
EdU 24h
Magnification
B
4 h
24h
Percent of GFP+EdU+ cells(0/000)
0.09
***
**
Control + Vehicle
DDC + Vehicle
DDC + GW4064
DDC + GW7647
C
EdU
BrdU
Merge

Figure S6 Labeling DNA in vivo by using EdU. (A) *Lgr5*-Cre^{ERT2}; Rosa26-mTmG mice fed with DDC diet or normal diet were injected intraperitoneally with 5 mg/kg of EdU in PBS. Livers were harvested, fixed, and sectioned 4 h or 24 h later. The images were overlays of GFP (Green) and EdU (Red) staining of the sectioned tissue. Scale bar represents 50 μm for 4 h and 24 h EdU staining, 20 μm for magnification. (B) Graphs showing percentages of $GFP^{+}EdU^{+}$ cell in the indicated groups. (C) Overlay of the BrdU and EdU images. Scale bars, 50 μm. Data were expressed as means ± SD, $^{**}p < 0.01$ and $^{***}p < 0.001$ were determined by one-way ANOVA.

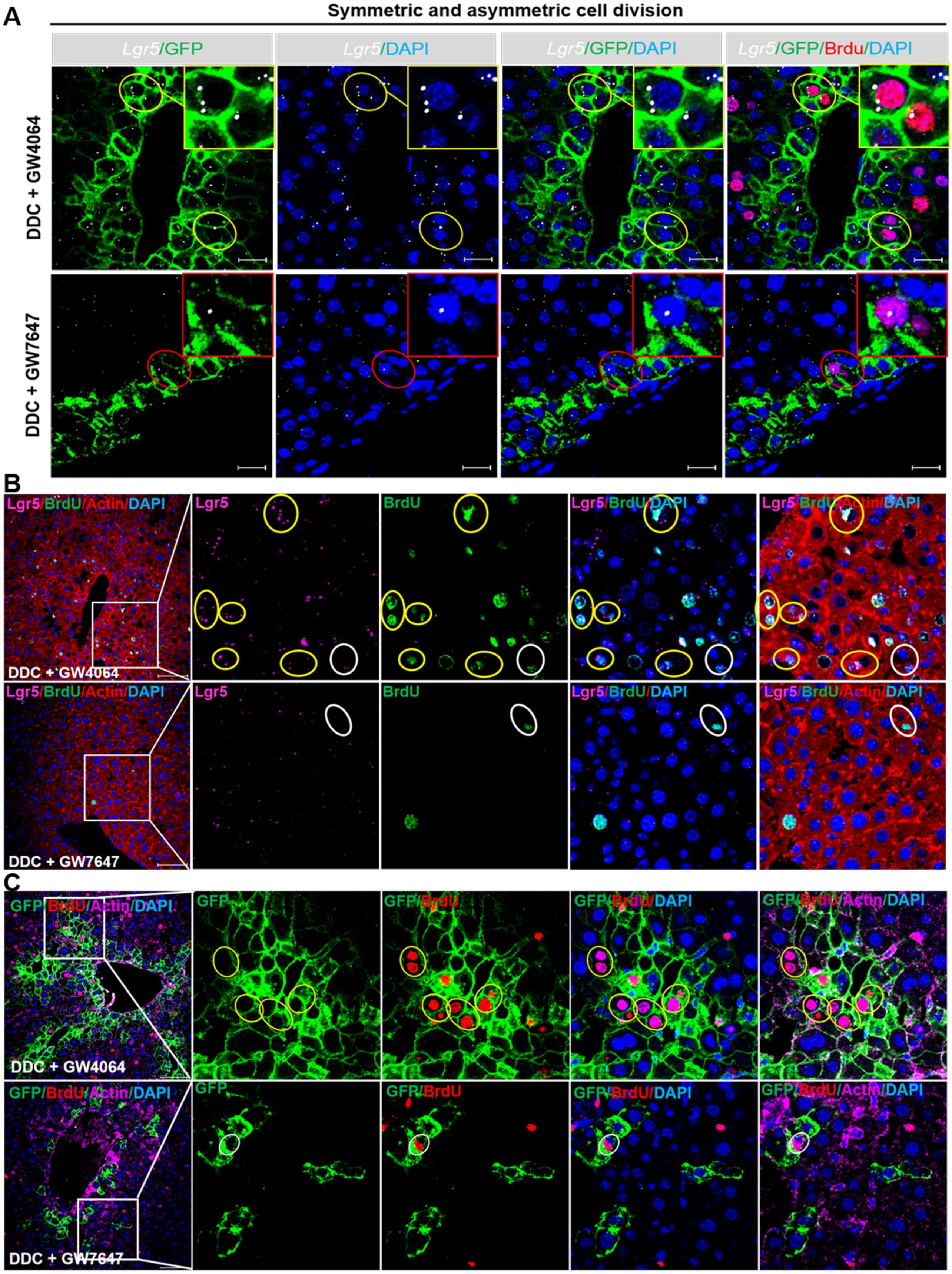

Figure S7 *Lgr5* symmetry or asymmetry division in GFP⁺ liver stem cell in livers of

DDC diet mice treated with GW4064 or GW7647. (A) ISH for *Lgr5* combined with IF for GFP and BrdU was performed to confirm *Lgr5* expression and *Lgr5*$^+$ stem cell proliferation. Yellow circle represents symmetric cell division (both of the daughter cells express *Lgr5*) and red circle represents asymmetric cell division (one stem cell with *Lgr5* expression and one differentiation cell without *Lgr5* expression). GFP in green, BrdU in red, *Lgr5* in white and DAPI in blue. Scale bars, 20 μm. (B) ISH for *Lgr5* combined with IF for BrdU and Actin (β-actin) was performed to confirm *Lgr5* expression and *Lgr5*$^+$ stem cell proliferation. Yellow circle represents symmetric cell division (*Lgr5 was* expressed in both of the daughter cells) and white circle represents asymmetric cell division (one stem cell with *Lgr5* expression and one differentiated cell without *Lgr5* expression). BrdU in green, Actin (β-actin) in red, *Lgr5* in red violet and DAPI in blue. Scale bars, 100 μm. (C) Multiple fluorochrome labeled antibody hybridization on the indicated liver samples. GFP in green, BrdU in red, Actin (β-actin) in red violet and DAPI in blue. Scale bars, 100 μm.

Supplemental Tables

Table S1. The primer sequences for QRT-PCR, EMSA, and ChIP

**Name**	**Application**	**Sequence (5'-3')**
H-*Cpt1a*-F	QRT-PCR	CATTCAGGCAGCAAGAGC
H-*Cpt1a*-R	QRT-PCR	CAGCAGCCGCCCATCATG
H-*FXR*-F	QRT-PCR	CCTCAACACTTTGCCTGTCTCCTG
H-*FXR*-R	QRT-PCR	GACATCAGCATCTCAGCGTGGTG
H-*Gapdh*-F	QRT-PCR	CTCTGGTAAAGTGGATATTG
H-*Gapdh*-R	QRT-PCR	GGTGGAATCATATTGGAAC
H-*Lgr5*-F	QRT-PCR	AGAATGGGGTTGGTTGCCAT

H-*Lgr5*-R	QRT-PCR	TTCACAGAGAACCCACGCTC
H-*PPARα*-F	QRT-PCR	CGGTGACTTATCCTGTGGTCC
H-*PPARα*-R	QRT-PCR	CCGCAGATTCTACATTCGATGTT
H-*Shp*-F	QRT-PCR	CCCAAGATGCTGTGACCTTT
H-*Shp*-R	QRT-PCR	CCAGAAGGACTCCAGACAGC
M-*36B4*-F	QRT-PCR	TGGAGACAAGGTGGGAGCC
M-*36B4*-R	QRT-PCR	CACAGACAATGCCAGGACGC
M-*Acox1*-F	QRT-PCR	CCTGATTCAGCAAGGTAGGG
M-*Acox1*-R	QRT-PCR	TCGCAGACCCTGAAGAAATC
M-*Bsep*-F	QRT-PCR	GTCTGACTCAGTGATTCTTCGC
M-*Bsep*-R	QRT-PCR	GAGCAATGCGCACACACTTC
M-*Cpt1a*-F	QRT-PCR	AGTGGCCTCACAGACTCCAG
M-*Cpt1a*-R	QRT-PCR	GCCCATGTTGTACAGCTTCC
M-*FXR*-F	QRT-PCR	TCCGGACATTCAACCATCAC
M-*FXR*-R	QRT-PCR	TCACTGCACATCCCAGATCTC
M-*Lgr5*-RT1-F	QRT-PCR	AGGCTGCCAAAAACTTCAGA
M-*Lgr5*-RT1-R	QRT-PCR	TCCATGCTAAGTTCAGAGATCG
M-*Lgr5*-RT2-F	QRT-PCR	CTGACTTTGAATGGTGCCTCG
M-*Lgr5*-RT2-R	QRT-PCR	ATGTCCACTACCGCGATTAC
M-*PPARα*-F	QRT-PCR	AGTTGCAGGAGGGGATTGT
M-*PPARα*-R	QRT-PCR	TGAAGGAGCTTTGGGAAGAG
M-Shp-F	QRT-PCR	CGATCCTCTTCAACCCAGATG
M-Shp-R	QRT-PCR	AGGGCTCCAAGACTTCACACA
M-*TNFα*-F	QRT-PCR	CATCTTCTCAAAATTCGAGTGACAA
M-*TNFα*-R	QRT-PCR	TGGGAGTAGACAAGGTACAACCC
Bio-*Lgr5*-probe-DR2-F	EMSA	AGGCAGGTGAAGAGGCAAGTT
Bio-*Lgr5*-probe-DR2-R	EMSA	AACTTGCCTCTTCACCTGCCT
Mut-*Lgr5*-probe-DR2-F	EMSA	AGGCATATGCGGATGCAAGTT
Mut-*Lgr5*-probe-DR2-R	EMSA	AACTTGCATCCGCATATGCCT

WT-*Lgr5*-probe-DR2-F	EMSA	AGGCAGGTGAAGAGGCAAGTT
WT-*Lgr5*-probe-DR2-R	EMSA	AACTTGCCTCTTCACCTGCCT
M-*Lgr5*-DR2-F	ChIP	CCCAACAAAAATCCATACTCGG
M-*Lgr5*-DR2-R	ChIP	CCTTTTAGTCCTAGGAGTGGTTC
M-*Gapdh*-F	ChIP	CGCAGTGAAAGCAGGAGACT
M-*Gapdh*-R	ChIP	ACCCCCTGTACCTCAGAAGG

H: Human M: Mouse F: Forward R: Reverse

Table S2. The probe information for RNAscope® assay

**Official symbol**	**Cat No.**	**Gene ID**	**Genbank nucleotide accession number**	**Channel**	**Detection Kit**
Lgr5	312171	14160	NM_010195.2	C3	RNAscope Multiplex Fluorescent Reagent Kit v2 (323100)
Hnf4α	497651	15378	NM_008261.3	C2	
Pck1	458021	18534	NM_011044.2	C1	
Gs	426231	14645	NM_008131.4	C1	
Positive probe (Mm)	320881	-	-	C1/C2/C3	
Negative probe	320871	-	-	C1/C2/C3	

References

[1] Liu D, He X, Qian P, Barker N, Trainor P, Clevers H, et al. Leucine-rich repeat-containing G-protein-coupled Receptor 5 marks short-term hematopoietic stem and progenitor cells during mouse embryonic development. J Biol Chem 2014;289(34):23809-16.

[2] Cao W, Chen K, Bolkestein M, Yin Y, Verstegen M, Bijvelds M, et al. Dynamics of proliferative and quiescent stem cells in liver homeostasis and injury. Gastroenterology 2017;153(4):1133-47.
